# Large Language Models and Artificial Neural Networks for Assessing 1-Year Mortality in Patients With Myocardial Infarction: Analysis From the Medical Information Mart for Intensive Care IV (MIMIC-IV) Database

**DOI:** 10.2196/67253

**Published:** 2025-05-12

**Authors:** Boqun Shi, Liangguo Chen, Shuo Pang, Yue Wang, Shen Wang, Fadong Li, Wenxin Zhao, Pengrong Guo, Leli Zhang, Chu Fan, Yi Zou, Xiaofan Wu

**Affiliations:** 1 Department of Cardiology Beijing Anzhen Hospital Capital Medical University Beijing China

**Keywords:** artificial neural network, large language model, myocardial infarction, prediction model, risk assessment

## Abstract

**Background:**

Accurate mortality risk prediction is crucial for effective cardiovascular risk management. Recent advancements in artificial intelligence (AI) have demonstrated potential in this specific medical field. Qwen-2 and Llama-3 are high-performance, open-source large language models (LLMs) available online. An artificial neural network (ANN) algorithm derived from the SWEDEHEART (Swedish Web System for Enhancement and Development of Evidence-Based Care in Heart Disease Evaluated According to Recommended Therapies) registry, termed SWEDEHEART-AI, can predict patient prognosis following acute myocardial infarction (AMI).

**Objective:**

This study aims to evaluate the 3 models mentioned above in predicting 1-year all-cause mortality in critically ill patients with AMI.

**Methods:**

The Medical Information Mart for Intensive Care IV (MIMIC-IV) database is a publicly available data set in critical care medicine. We included 2758 patients who were first admitted for AMI and discharged alive. SWEDEHEART-AI calculated the mortality rate based on each patient’s 21 clinical variables. Qwen-2 and Llama-3 analyzed the content of patients’ discharge records and directly provided a 1-decimal value between 0 and 1 to represent 1-year death risk probabilities. The patients’ actual mortality was verified using follow-up data. The predictive performance of the 3 models was assessed and compared using the Harrell C-statistic (C-index), the area under the receiver operating characteristic curve (AUROC), calibration plots, Kaplan-Meier curves, and decision curve analysis.

**Results:**

SWEDEHEART-AI demonstrated strong discrimination in predicting 1-year all-cause mortality in patients with AMI, with a higher C-index than Qwen-2 and Llama-3 (C-index 0.72, 95% CI 0.69-0.74 vs C-index 0.65, 0.62-0.67 vs C-index 0.56, 95% CI 0.53-0.58, respectively; all *P*<.001 for both comparisons). SWEDEHEART-AI also showed high and consistent AUROC in the time-dependent ROC curve. The death rates calculated by SWEDEHEART-AI were positively correlated with actual mortality, and the 3 risk classes derived from this model showed clear differentiation in the Kaplan-Meier curve (*P*<.001). Calibration plots indicated that SWEDEHEART-AI tended to overestimate mortality risk, with an observed-to-expected ratio of 0.478. Compared with the LLMs, SWEDEHEART-AI demonstrated positive and greater net benefits at risk thresholds below 19%.

**Conclusions:**

SWEDEHEART-AI, a trained ANN model, demonstrated the best performance, with strong discrimination and clinical utility in predicting 1-year all-cause mortality in patients with AMI from an intensive care cohort. Among the LLMs, Qwen-2 outperformed Llama-3 and showed moderate predictive value. Qwen-2 and SWEDEHEART-AI exhibited comparable classification effectiveness. The future integration of LLMs into clinical decision support systems holds promise for accurate risk stratification in patients with AMI; however, further research is needed to optimize LLM performance and address calibration issues across diverse patient populations.

## Introduction

Although the epidemiologic characteristics and treatments of acute myocardial infarction (AMI) have changed substantially over the past 3-4 decades, the estimated mortality rate within the first year following AMI remains high, at 15%-20% [1]. Thus, prompt and accurate risk prediction and stratification are crucial for cardiovascular risk assessment and management after MI. Among the many models for risk prediction, artificial intelligence (AI)–based predictive models and their subdiscipline, machine learning (ML), have been an active field of research, including in cardiovascular medicine [2]. Mohammad and his colleagues [3] recently developed an artificial neural network (ANN) algorithm, which consists of 21 easily obtainable variables to predict mortality and hospitalization for heart failure within 1 year after MI. This ANN model was trained and tested in the SWEDEHEART (Swedish Web System for Enhancement and Development of Evidence-Based Care in Heart Disease Evaluated According to Recommended Therapies) registry, termed SWEDEHEART-AI, and had been well validated in the Western Denmark Heart Registry. It also correctly identified outcomes better than popular risk scores, such as the Global Registry of Acute Coronary Events (GRACE) 2.0.

However, the calibration performance of SWEDEHEART-AI in non-Western populations has not been fully validated and may lead to prediction bias due to differences in population characteristics. Additionally, SWEDEHEART-AI requires doctors to collate the input of 21 variables to obtain the predicted probability. When faced with large amounts of real-world data, such as electronic health records (EHRs), extracting relevant characteristics can be difficult and time-consuming, yet it is essential for evaluating disease progression. More importantly, SWEDEHEART-AI was unable to assess the prognostic impact of clinical characteristics beyond these 21 variables (eg, N-terminal pro-B-type natriuretic peptide or the presence of coronary artery bypass grafting), which limited its ability to provide individualized predictions for different patients. Newly developed large language models (LLMs) offer an exciting approach to solving clinical text processing challenges, as they can respond to free-text inquiries without specialized training for the relevant tasks [4]. LLMs are transformer-based architectures that enable the understanding, processing, and generation of large-scale natural language text by scaling up model size, pretrained corpora, and computational resources [5]. As LLMs evolve in size, their improving capabilities have radically altered natural language processing [6]. Some LLMs have already been applied in health care and have played a remarkable role [4]. Qwen-2 and Llama-3 are new open-source LLMs developed by Alibaba Cloud (Alibaba Group Holding Limited) and Meta (Meta Platforms, Inc), respectively, for understanding and analyzing natural language input to assist users across various domains and tasks [7,8]. For Qwen-2, all instruction-tuned models have been trained on 32,000-length contexts and extrapolated to longer context lengths using techniques such as Yet Another RoPE Extension or Dual Chunk Attention. For more details, see [9]. For Llama-3, its team innovated on the approach to instruction tuning to fully unlock the potential of pretrained models in chat use cases. The approach to posttraining combines supervised fine-tuning, rejection sampling, proximal policy optimization, and direct preference optimization. Llama-3 is pretrained on over 15 trillion tokens, all collected from publicly available sources. For more details, please see [10].

Therefore, we aimed to compare the performance of SWEDEHEART-AI, Qwen-2, and Llama-3 in predicting 1-year all-cause mortality in patients with AMI using the Medical Information Mart for Intensive Care IV (MIMIC-IV, version 2.2) database, and to explore whether LLMs can provide critical risk prediction and influence clinical decisions for patients with cardiovascular issues. We hypothesize that LLMs may perform on par with or inferior to the disease-specific model, SWEDEHEART-AI, in AMI mortality prediction, due to the complexity of medical text, insufficient training of LLMs on domain-specific knowledge, and the lack of fine-tuning for the clinical task. However, the potential future assistance of LLMs in the medical field should not be underestimated.

## Methods

### Study Design and Populations

**Figure 1:**
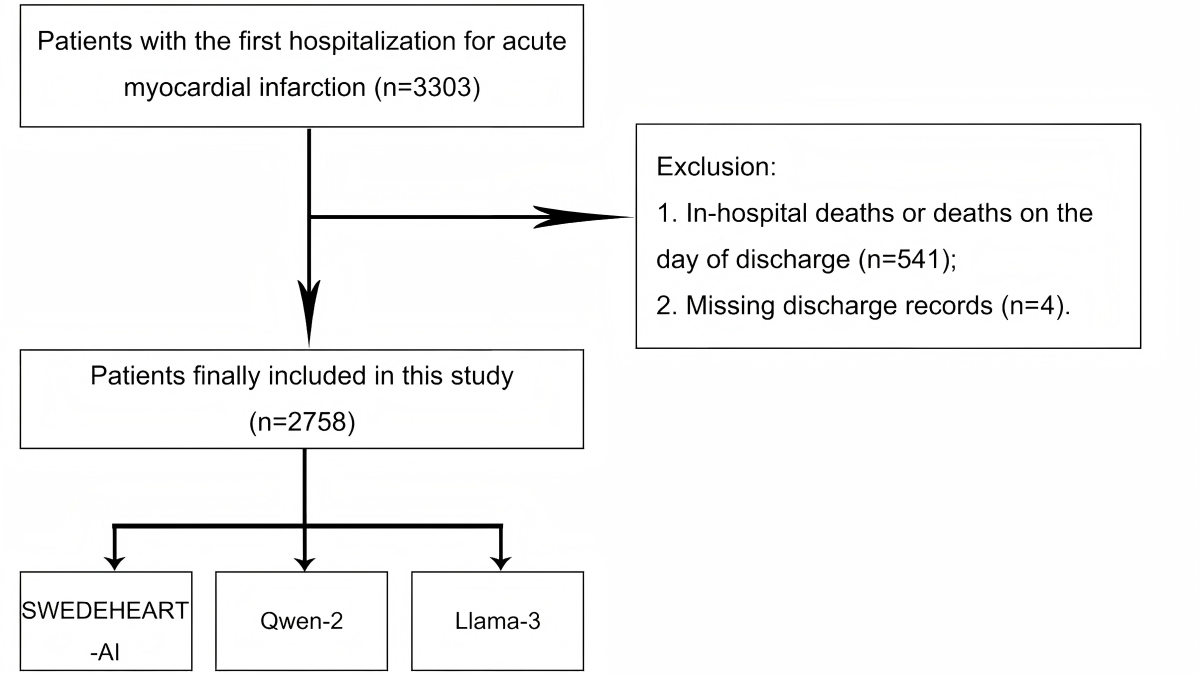
Flowchart for inclusion and exclusion. SWEDEHEART: Swedish Web System for Enhancement and Development of Evidence-Based Care in Heart Disease Evaluated According to Recommended Therapies.

The MIMIC-IV database is a publicly available EHR data set that provides clinical data in critical care medicine [11]. Among the 3303 patients admitted for the first time for AMI in the MIMIC-IV database, we excluded 541 patients who had in-hospital deaths (hospital_expire_flag=1) or died on the day of discharge, and further excluded 4 patients with missing discharge records. Finally, 2758 patients admitted for AMI and discharged alive comprised our analysis cohort (Figure 1). This work follows the TRIPOD (Transparent Reporting of a Multivariable Prediction Model for Individual Prognosis or Diagnosis) statement [12].

### Ethics Considerations

The study was conducted in accordance with the guidelines of the Helsinki Declaration. The Review Committee of the Massachusetts Institute of Technology and Beth Israel Deaconess Medical Center approved access to the MIMIC-IV database. Two of the authors (BS and LC) fulfilled the database access request and were responsible for extracting data related to hospitalized patients using SQL. All these data were deidentified; therefore, the study was exempt from ethical approval and informed consent requirements.

### Outcome of Interest and Risk Probability Acquisition

The end point of interest was all-cause death within 1 year after AMI. The patients’ actual 1-year mortality was verified using follow-up data from the MIMIC-IV database. The SWEDEHEART-AI–derived risk probabilities for patients were obtained following the instructions in the original article using STATA (StataCorp), with 21 variables, including age, gender, previous medical history (hypertension, diabetes, chronic heart failure, history of myocardial infarction, and stroke), prior medications (aspirin, β-blockers, angiotensin-converting enzyme inhibitors or angiotensin II receptor blockers, and antidiabetic and lipid-lowering agents), in-hospital features (ST-elevation myocardial infarction [STEMI], non-STEMI [NSTEMI], coronary angiography, heart rate, and systolic blood pressure), left ventricular ejection fraction, and medications at discharge (P2Y12 inhibitors, β-blockers, and angiotensin-converting enzyme inhibitors or angiotensin II receptor blockers) [3], all of which can be extracted from the MIMIC-IV database. The note module in the database contains deidentified free-text EHR. We input patients’ discharge records into Qwen-2 and Llama-3 with the instruction: “Please select the number from 0, 0.1, 0.2, 0.3, 0.4, 0.5, 0.6, 0.7, 0.8, 0.9, and 1 that you believe is closest to the patient’s 1-year all-cause mortality risk based on the discharge record, and output only your numerical answer, without analysis.” Qwen-2 and Llama-3 analyzed the content of the EHR and returned the risk probability values. Ollama [13] was used to deploy Qwen-2.0 7B and Llama-3.0 8B on an RTX 4090 (Nvidia Corporation) laptop for offline computing. In detail, we first visited the Ollama website to download and install the Ollama framework according to the system type. Using the Ollama client or command-line tool, we located and downloaded the Qwen-2.0 7B and Llama-3.0 8B models from their model repositories to a locally specified directory. Next, we specified the use of local model paths in the Ollama configuration and ensured that the laptop was offline to avoid the risk of data leakage.

### Statistical Analysis

Continuous variables were represented as medians with IQRs, while categorical variables were represented as frequencies with percentages. Regarding the handling of missing values, if any of the 21 variables required for SWEDEHEART-AI were missing, we followed the approach described in the original article: missing values for categorical variables were converted into a separate “missing” category, which was handled by the ANN. LLMs typically handle missing values in linguistic texts using the following methods: replacing the missing part with a special token, dynamically managing missing values through context dependency, and using the attention mechanism to address missing values. The discriminative ability was assessed using the Harrell C-statistic (C-index) and the time-dependent receiver operating characteristic (ROC) curve. The agreement between observed and predicted event rates was evaluated using calibration plots in deciles of predicted risk. Calibration was considered optimal when the calibration curve was close to the diagonal line, reflected by an observed-to-expected ratio near 1 [14], and the Hosmer-Lemeshow test showed a *P* value greater than .05. The precision-recall curve was also used to compare different models in the highly skewed data set, addressing the optimism of the ROC curve [15]. To examine whether the SWEDEHEART-AI model correlated with all-cause death, Kaplan-Meier curves were stratified by tertiles, and strata were compared using the log-rank test at 1 year. The restricted cubic spline with 3 knots was used to model the relationship between SWEDEHEART-AI and 1-year mortality flexibly. Potential nonlinearity was tested using the likelihood ratio test, comparing a model with only linear terms with a model containing both linear and cubic spline terms [16]. We compared the ability of the SWEDEHEART-AI score to categorize the risk of all-cause mortality with that of LLMs using continuous and categorical net reclassification improvement (NRI) and integrated discrimination improvement (IDI). Positive NRI values indicated more accurate reclassification, while negative values indicated incorrect reclassification. Positive IDI values indicated improved discrimination, whereas negative values indicated that the model did not improve. Decision curve analysis (DCA) was used to assess net benefits and clinical utility [17]. A 2-sided *P* value less than .05 was considered statistically significant. Data analysis was performed using R version 4.4.1 (R Foundation).

## Results

### Baseline Characteristics

This study included 2758 patients who were admitted to the hospital for the first time for AMI and were discharged alive from the MIMIC-IV database (Figure 1). The age and gender composition in our cohort were similar to that of the SWEDEHEART-AI cohort (Table 1). Patients in our cohort had a higher proportion of diabetes mellitus (1068/2758, 38.72% vs 23,506/111,558, 21.07%) and chronic heart failure (1297/2758, 47.03% vs 8199/111,558, 7.35%), but a lower proportion of prior hypertension (1120/2758, 40.61% vs 56,086/111,558, 50.28%) and a history of MI (289/2758, 10.48% vs 23,153/111,558, 20.75%) compared with the SWEDEHEART-AI cohort. The majority of our patients (1580/2758, 57.29%) were diagnosed with STEMI, while the remaining patients were classified as non-STEMI (NSTEMI). The SWEDEHEART-AI cohort was predominantly composed of NSTEMI cases (74,661/111,558, 66.93%). In our cohort, the proportion undergoing coronary angiography was much lower (579/2758, 20.99% vs 89,045/111,558, 79.82%), as was the systolic blood pressure (116 mm Hg vs 149 mm Hg). The proportion of patients in our cohort with a left ventricular ejection fraction greater than 50% was lower (601/2758, 21.79% vs 51,884/111,558, 46.51%), while the proportion with unknown left ventricular ejection fraction was higher (1587/2758, 57.54% vs 23,996/111,558, 21.51%). The proportions of patients receiving all discharge medications were lower in our cohort. By 1 year, 475 patients (475/2758, 17.2%) had died from any cause in our cohort. In the combined SWEDEHEART-AI cohort (111,558 in training cohorts and 27,730 in testing cohorts), the 1-year mortality rate was less than 10% (13,407/139,288, 9.63%).

**Table 1:** Baseline demographic factors and variables^a^.

Demographics		SWEDEHEART-AI^b^ (n=111,558)	MIMIC-IV^c^ (n=2758)
Age (years), median (range)		71.0 (62.0-80.0)	70.0 (61.0-80.0)
**Sex, n (%)**			
	Men	72,977 (65.42)	1777 (64.43)
	Women	38,581 (34.58)	981 (35.57)
**Medical history, n (%)**			
	Hypertension	56,086 (50.28)	1120 (40.61)
	Diabetes	23,506 (21.07)	1068 (38.72)
	Chronic heart failure	8199 (7.35)	1297 (47.03)
	History of myocardial infarction	23,153 (20.75)	289 (10.48)
	Stroke	9237 (8.28)	227 (8.23)
**In-hospital characteristics**			
	STEMI^d^, n (%)	36,897 (33.07)	1580 (57.29)
	NSTEMI^e^, n (%)	74,661 (66.93)	1178 (42.71)
	Coronary angiography, n (%)	89,045 (79.82)	579 (20.99)
	Heart rate (beats/min), median (IQR)	79 (67-92)	80 (72-91)
	Systolic blood pressure (mm Hg), median (IQR)	149 (130-167)	116 (106-124)
	Creatinine (μmol/L), median (IQR)	83 (69-100)	88 (71-133)
**Ejection fraction, n (%)**			
	≥50%	51,884 (46.51)	601 (21.79)
	40%-49%	19,071 (17.10)	276 (10.01)
	30%-39%	11,745 (10.53)	195 (7.07)
	<30%	4862 (4.36)	99 (3.59)
	Unknown	23,996 (21.51)	1587 (57.54)
**Discharge medications, n (%)**			
	P2Y12 inhibitor	90,337 (80.98)	581 (21.07)
	β-blockers	98,472 (88.27)	2358 (85.50)
	Angiotensin-converting enzyme inhibitors or angiotensin II receptor blockers	85,984 (77.08)	1490 (54.02)

^a^The baseline demographic variables of the SWEDEHEART-AI cohort were from the training set.

^b^SWEDEHEART: Swedish Web System for Enhancement and Development of Evidence-Based Care in Heart Disease Evaluated According to Recommended Therapies.

^c^MIMIC: Medical Information Mart for Intensive Care.

^d^STEMI: ST-elevation myocardial infarction.

^e^NSTEMI: non–ST-elevation myocardial infarction.

### Discrimination

**Figure 2:**
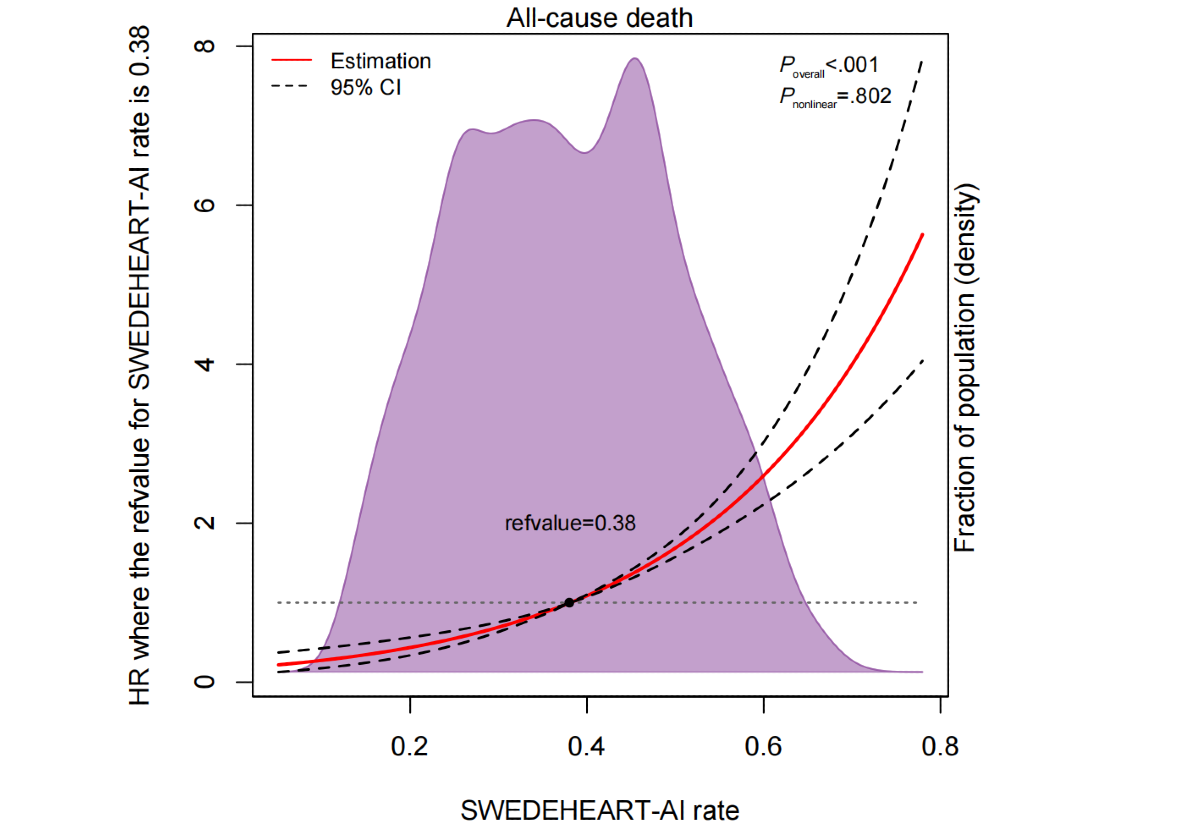
Restricted cubic spline analysis for SWEDEHEART-AI and 1-year mortality. SWEDEHEART: Swedish Web System for Enhancement and Development of Evidence-Based Care in Heart Disease Evaluated According to Recommended Therapies.

The restricted cubic spline analysis showed a positive relationship between the SWEDEHEART-AI estimated risk probabilities and all-cause mortality, with the reference value for the SWEDEHEART-AI rate at 0.38 (*P*_overall_<.001 and *P*_nonlinearity_=.80; Figure 2). When patients were stratified by tertiles of the SWEDEHEART-AI score in the Kaplan-Meier curve, those with a high-risk SWEDEHEART-AI score had a higher cumulative incidence of mortality compared with low-risk patients (all log-rank *P*<.001; Multimedia Appendix 1).

**Figure 3:**
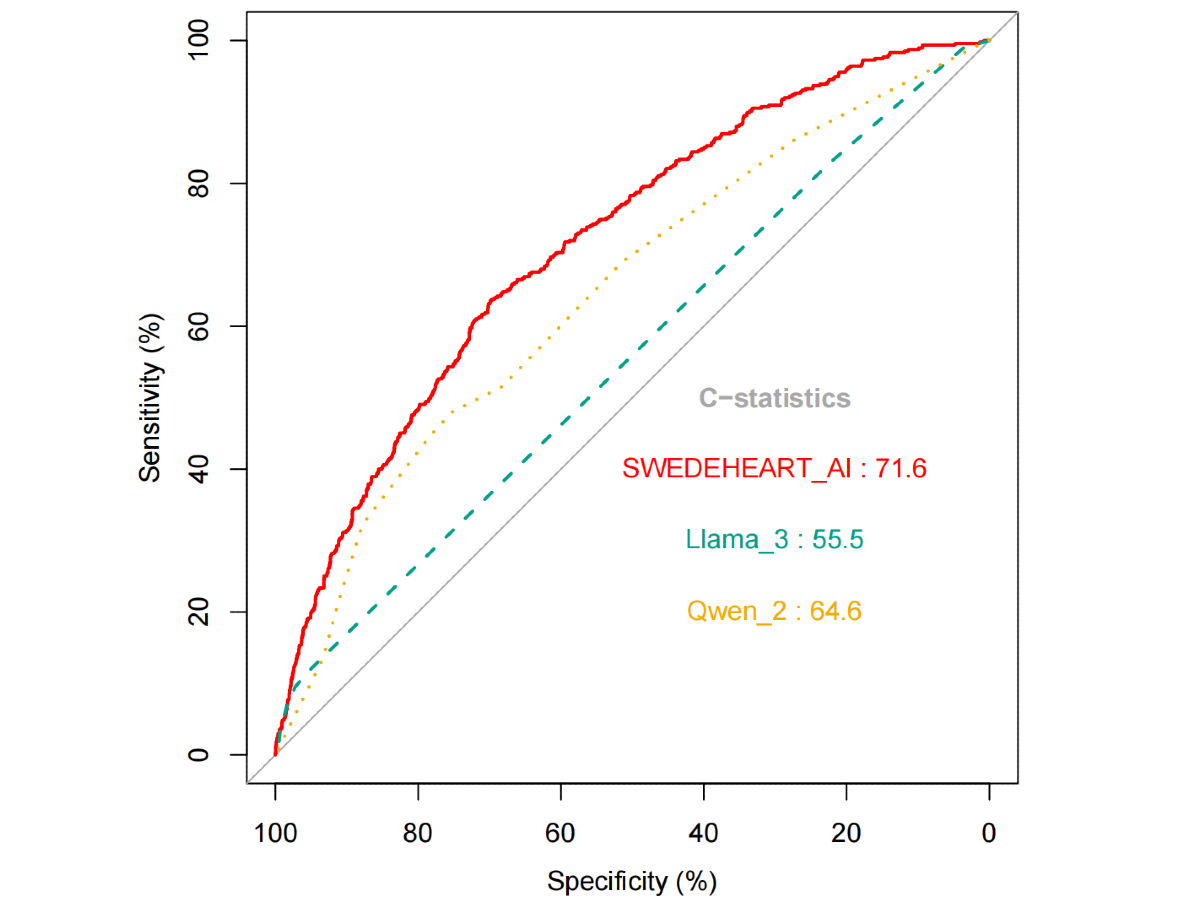
Receiver operating characteristics curves for all-cause mortality. SWEDEHEART: Swedish Web System for Enhancement and Development of Evidence-Based Care in Heart Disease Evaluated According to Recommended Therapies.

The SWEDEHEART-AI model displayed relatively strong discrimination in predicting 1-year mortality in patients with AMI, with a higher C-index than Qwen-2 and Llama-3 (C-index 0.72, 95% CI 0.69-0.74 vs C-index 0.65, 95% CI 0.62-0.67 vs C-index 0.56, 95% CI 0.53-0.58, respectively; all *P*<.001 for both comparisons; also see Figure 3 and Table 2). Using the maximal Youden index cutoff (0.47), 1897 out of 2758 (68.8%) patients were correctly classified for 1-year mortality by SWEDEHEART-AI, corresponding to a sensitivity of 63.6%, specificity of 69.9%, negative predictive value (NPV) of 90.2%, and positive predictive value (PPV) of 30.5% (Table 2). Qwen classified 1946 out of 2758 (70.56%) patients correctly for 1-year mortality, with a sensitivity of 48.0%, specificity of 75.3%, NPV of 87.4%, and PPV of 28.8%. Llama classified 2239 out of 2758 (81.18%) patients correctly, with a sensitivity of 11.4%, specificity of 95.7%, NPV of 83.8%, and PPV of 35.5%.

SWEDEHEART-AI also achieved a higher area under the ROC curve (AUROC) in the time-dependent ROC analysis and demonstrated stable predictive performance over the 1-year period (Multimedia Appendix 2). Additionally, SWEDEHEART-AI had a higher area under the precision-recall curve compared with Qwen-2 and Llama-3 (0.347 vs 0.227 vs 0.270, respectively; Multimedia Appendix 3).

**Table 2:** Model performance for predicting 1-year all-cause death risk^a^.

Index	SWEDEHEART-AI^b^	Qwen-2	Llama-3
C-statistic/C-index (95% CI)	0.72 (0.69-0.74)	0.65 (0.62-0.67)	0.56 (0.53-0.58)
Best cutoff value	0.47	0.55	0.45
Sensitivity	63.58	48.00	11.37
Specificity	69.91	75.25	95.71
Negative predictive value	90.22	87.43	83.84
Positive predictive value	30.54	28.75	35.53
False-positive rate	30.09	24.75	4.29
False-negative rate	36.42	52.00	88.63
False discovery rate	69.46	71.25	64.47
Accuracy	68.82	70.56	81.18
Youden index	133.49	123.25	107.08

^a^*P* values of the Delong test are <.001 for SWEDEHEART-AI versus Qwen-2, SWEDEHEART-AI versus Llama-3, and Qwen-2 versus Llama-3.

^b^SWEDEHEART: Swedish Web System for Enhancement and Development of Evidence-Based Care in Heart Disease Evaluated According to Recommended Therapies.

### Calibration

**Figure 4:**
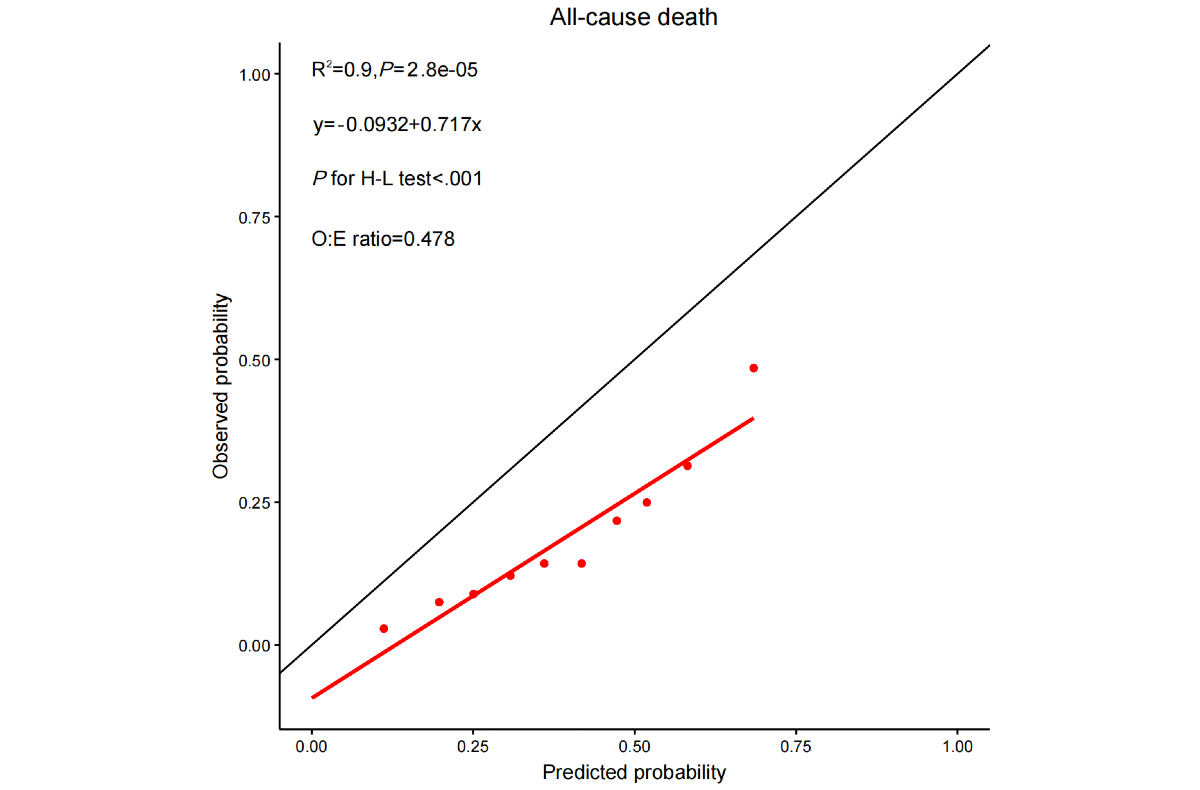
Calibration plots of the SWEDEHEART-AI score. H-L: Hosmer-Lemeshow; O:E: observed-to-expected ratio; SWEDEHEART: Swedish Web System for Enhancement and Development of Evidence-Based Care in Heart Disease Evaluated According to Recommended Therapies.

SWEDEHEART-AI showed poor calibration for predicting all-cause mortality (*P* value for the Hosmer-Lemeshow test <.001), when stratified by deciles of event probability based on the SWEDEHEART-AI score (Figure 4). The calibration curve for 1-year mortality fell below the perfect calibration line, with an observed-to-expected ratio of 0.478. The calibration column also indicated that, despite the deciles of predicted risk, SWEDEHEART-AI consistently overestimated the risk of death (Multimedia Appendix 4).

SWEDEHEART-AI showed excellent improvement in predicting all-cause mortality in terms of NRI (0.4328, 95% CI 0.3391-0.5265, *P*<.001) and IDI (0.1007, 95% CI 0.0830-0.1183, *P*<.001) compared with Llama-3 (Multimedia Appendix 5). However, SWEDEHEART-AI did not show significant improvement for all-cause mortality in terms of NRI (–0.0247, 95% CI –0.1233 to 0.0738, *P*=.62) and IDI (–0.0089, 95% CI –0.0393 to 0.0215, *P*=.57) compared with Qwen-2 (Multimedia Appendix 5).

### Decision Curve Analysis

The DCA demonstrates the clinical utility of each model, showing the potential threshold for outcome risk (x-axis) and the net benefit of using the model (y-axis), assuming that no patient will experience an event. Compared with the LLMs, the net benefit of using SWEDEHEART-AI as a decision threshold was consistently positive and large over a wide range of 1-year mortality risks (Multimedia Appendices 6 and Multimedia Appendix 7). The net benefit curve for Qwen-2 and Llama-3 nearly overlapped with the treat-all curve. For SWEDEHEART-AI, the DCA showed a consistent positive net benefit for decision thresholds below 19% for 1-year mortality risk. For instance, at a 15% risk threshold, 39.3 per 1000 patients achieved a better net benefit compared with the treat-none scenario, 13.2 per 1000 patients benefited compared with the treat-all scenario, 12.8 per 1000 patients benefited compared with Qwen-2, and 13.1 per 1000 patients benefited compared with Llama-3 (Multimedia Appendix 7).

## Discussion

### Principal Findings

Our study is the first to explore the predictive performance of the ANN model, represented as SWEDEHEART-AI, and LLMs, represented as Qwen-2 and Llama-3, for 1-year all-cause mortality after MI using a critical care database. The main results of our study are summarized as follows: (1) Of the 2758 individuals, SWEDEHEART-AI, Qwen, and Llama correctly classified 1897 (68.78%), 1946 (70.56%), and 2239 (81.18%) for 1-year mortality, respectively; (2) SWEDEHEART-AI demonstrated significant discrimination in predicting 1-year all-cause mortality in patients with AMI, with a higher C-index than Qwen-2 and Llama-3 (0.72 vs 0.65 vs 0.56, respectively); (3) the risk probabilities estimated by SWEDEHEART-AI were positively correlated with actual all-cause mortality, but it consistently overestimated mortality risk with poor calibration; (4) SWEDEHEART-AI showed significant improvement over Llama-3, but no significant improvement over Qwen-2; (5) SWEDEHEART-AI also provided better clinical utility at thresholds of less than 19% mortality risk, as assessed by the DCA, while Qwen-2 and Llama-3 showed relatively weak clinical utility. Therefore, this study provides new insights for clinical physicians by introducing SWEDEHEART-AI and potential LLMs for predicting the risk of mortality after AMI, thereby offering improved decision support for critically ill patients with AMI.

### Comparison With Prior Work

AMI is the leading cause of death worldwide, contributing significantly to the global disease burden, with higher hospitalization and treatment costs [18]. The Western Denmark Heart Registry found that the 1-year mortality rate in patients with STEMI treated with primary percutaneous coronary intervention decreased substantially from 10.8% in 2003-2006 to 7.7% in 2015-2018. However, enhanced care for AMI remains necessary [19]. Therefore, the application of emerging AI for accurate cardiovascular risk prediction and stratification is a promising research direction with significant clinical value [20]. LLMs, as transformer-based architectures, excel in understanding language context and can be efficiently trained on vast amounts of unlabeled data. However, high-performance LLMs such as OpenAI’s ChatGPT and Google Bard are not open source, requiring patient data to be transmitted to their platforms for analysis, raising security concerns [21]. By contrast, Qwen-2 and Llama-3 are open-source, online LLMs that have garnered significant attention in various fields, including disease management and clinical decision-making [7].

Using different AI models in various scenarios may yield different results, and this is worth exploring in depth across domains. One study [22] evaluated ChatGPT’s ability to predict the progression of ocular hypertension to glaucoma. The results indicated that ChatGPT-4.0 predicted the transition to glaucoma 1 year before onset, with an accuracy of 75% and an AUROC of 0.67. This was an improvement over ChatGPT-3.5, which had an accuracy of 61% and an AUROC of 0.62 [22]. The value of another LLM, Vicuna-13B, in annotating radiological reports in the MIMIC-CXR data set (which includes the patient’s chest x-ray information) has been investigated, showing an AUROC of 0.84 [23]. However, LLMs are prone to hallucinations—referring to the AI’s ability to generate answers that seem plausible but may be incorrect or nonsensical—along with poor performance in complex reasoning, a tendency to perpetuate bias, and randomness [6]. One study applied an LLM to diagnosis-related groups (DRGs), fine-tuned to MIMIC-IV discharge EHRs, to improve the efficiency of DRG allocation. It proposed the DRG-Llama-7B model, which achieved an AUROC of 0.986 [24]. These studies suggest the potential value of using multimodal data combined with active learning to develop LLMs tailored to specific clinical departments in the future.

Increasingly, AI research has focused on prognosis following MI. We can confidently say that AI holds great promise for forecasting outcomes, as it can identify nonlinear correlations and self-learn from the vast amount of data generated [25]. One study used the Korean AMI Registry data set to select hyperparameter ranges from 4 different ML models to predict 1-year mortality. The AUROC of the applied ML algorithms improved, on average, by 0.08 compared with GRACE, with the gradient boosting machine and deep neural network achieving the highest AUROC of 0.898. These models also differed in terms of the main prognostic factors [26]. Another study using this cohort applied an ML model that achieved an AUROC of 0.918 [27], highlighting how different hyperparameters can impact model performance. By contrast, a study using the American College of Cardiology Chest Pain-MI Registry found that none of the tested ML models significantly improved the discrimination of in-hospital death after AMI. However, XG-Boost and meta-classifier models (rather than ANN) were able to better discriminate risk in high-risk populations compared with logistic regression [28]. The use of the ML-based Prediction of Adverse Events Following Acute Coronary Syndrome (PRAISE) score to predict all-cause mortality, MI, and hemorrhage after acute coronary syndrome demonstrated accurate discriminatory ability that can aid clinical decision-making [29]. However, in a real Asian population undergoing percutaneous coronary intervention for acute coronary syndrome, the PRAISE score showed limited potential, with C-index for death, MI, and major hemorrhage of 0.75, 0.61, and 0.62, respectively. The DCA showed that the PRAISE score provided a slightly higher net benefit (5%-10%) for the 1-year risk of death compared with the GRACE score [30]. Therefore, prediction models need to be readjusted for different populations.

Many AI models have not been disclosed for external validation, which limits their generalizability. The TRIPOD checklist [12] requires authors to present complete predictive models for individual predictions. However, the inherent complexity of ML and other advanced algorithms (often referred to as the “black box”) complicates risk calculation and external validation [31]. The SWEDEHEART-AI, based on the ANN model, overcame the “black box” challenge. Another similar ANN model was trained on risk factors such as systolic blood pressure, hemoglobin, and corrected QT interval, demonstrating higher accuracy (92.86%) in identifying patients with NSTEMI [32]. However, LLMs such as Qwen-2 and Llama-3 are difficult to interpret, as their internal workings are not transparent to the user. This lack of transparency can be problematic in applications that require high levels of interpretability and verifiability. Fortunately, several approaches and techniques can enhance the interpretability and transparency of ML models, such as feature importance, local interpretation using Shapley additive explanations, and global interpretation.

Extracting features from vast EHRs for risk prediction is both meaningful and challenging. Therefore, developing or leveraging existing AI and LLMs can serve as virtual clinical supporters [33]. Established advanced methods for textual data extraction include supervised learning techniques and pretrained models such as Bidirectional Encoder Representations from Transformers (BERT) [8]. However, low-parameter LLMs (around 10 billion parameters) often require multisystem support (eg, fine-tuning or complex reasoning chains) when addressing tasks in specialized scenarios with various uncommon issues, including feature illusions [34]. One study explored the capacity of deep learning methods to forecast prospective diseases based on EHRs, which were fed into a bidirectional gated recurrent unit model to predict the likelihood of MI occurring within the next 3 years. Expanding the scope of the data, such as incorporating a broader range of health determinants, could increase the AUROC to 0.94 for predicting MI [33]. Another study used a novel modular LLM pipeline, which enables the semantic extraction of features from patients’ admission EHR. The pipeline, using a low-parameter LLM (Qwen-14B-Chat), achieved accuracy and precision rates of 95.52% and 92.93%, respectively [8].

Our study found that although Llama-3 had the highest accuracy (81.2%), other performance metrics, such as AUROC and net benefit, were relatively poor. The data set used had a classification imbalance, so accuracy alone was not a reliable indicator of model performance. Our cohort had a 17.2% mortality rate (475/2758 deaths vs 2283 survivors). Llama-3 achieved high specificity (95.7%) but very low sensitivity (11.4%), meaning it correctly identified most survivors (true negatives) but missed 88.6% of the deaths (false negatives). In imbalanced data sets, models that prioritize specificity can inflate accuracy by favoring the majority class (survivors). However, this does not accurately reflect the model’s utility for predicting the critical minority class (deaths). The C-index measures how well a model ranks patients by risk. A low C-index (0.56) indicates that Llama-3 struggles to distinguish between patients who die and those who survive. Its high accuracy is due to correctly classifying survivors (the majority class), but it fails to meaningfully stratify risk or identify high-risk patients, which is essential for clinical decision-making. While Llama-3’s accuracy might seem impressive, its poor sensitivity and discriminative power (C-index) make it clinically inadequate for mortality prediction. In imbalanced settings, metrics such as C-index, AUROC, and sensitivity are more informative, as they prioritize identifying true positives (deaths) over inflating accuracy through true negatives (survivors). This phenomenon suggests that relying solely on accuracy may mislead model evaluation, and a more comprehensive assessment should include area under the curve and NPV indicators.

Similarly, a study evaluated the clinical accuracy of GPT and Llama-2 in making initial diagnoses, recommending examination procedures, and suggesting therapies for about 100 cases from various clinical sections. GPT-4 performed the best, with Llama-2 performing slightly worse [35]. However, the commercial version of Llama has shown increasing promise in responding to medical inquiries in 2 consecutive major releases. To address specific needs related to data privacy and training transparency, open-source LLMs may be a more viable choice.

Accurate prediction of future mortality can help doctors identify patients who would benefit from intensive therapy. The SWEDEHEART-AI provided the original model for validation, and it performed relatively well, achieving the highest C-index and area under the precision-recall curve compared with the LLMs. We suggest that this superiority may primarily stem from the aspects described in Textbox 1.

**Aspects suggesting the superiority of SWEDEHEART-AI.**

1. Use of clinically relevant variables

The SWEDEHEART-AI model was built based on 21 clinically relevant variables, carefully selected to comprehensively reflect the clinical characteristics and risk factors of patients with acute myocardial infarction. By contrast, large language models (eg, Qwen-2 and Llama-3) predict risk primarily through the natural language processing of patient discharge records. While they can process large amounts of textual information, they may not be as accurate as SWEDEHEART-AI in extracting key clinical variables. This difference may result in SWEDEHEART-AI exhibiting greater accuracy and stability in predicting all-cause mortality over a 1-year period.

2. Model structure and optimization

SWEDEHEART-AI utilizes a multilayered artificial neural network with a backpropagation algorithm. The artificial neural network mimics the structure of the human brain, consisting of interconnected artificial neural network nodes organized into an input layer, multiple hidden layers, and an output layer. This structure allows for backpropagation, enabling self-learning and model optimization. To improve accuracy, the algorithm was fine-tuned by increasing the number of hidden layers and training iterations until no further improvement was observed. Ultimately, the SWEDEHEART-AI model used 10 hidden layers. Although large language models excel in natural language processing, they may struggle with clinical prediction tasks without specific tuning. Our findings provide an important reference for future research.

The sensitivity of SWEDEHEART-AI was the highest among our 3 models but still not optimal. The SWEDEHEART-AI score overestimated all-cause death in all deciles, although it had the highest NPV. This overestimation may be attributed to our cohort having a higher mortality rate (475/2758, 17.22% vs 13,407/139,288, 9.63%) compared with the SWEDEHEART registry. Such overestimation could have negative implications for clinical applications, potentially leading to overintervention. If physicians rely solely on the predictive results of the SWEDEHEART-AI model, there is a risk of overintervening in low-risk patients, which could increase the health care burden and expose patients to unnecessary risks. Therefore, it is essential to consider prognosis within the context of clinical experience and real-life situations. For low-risk patients, follow-up care by a cardiac rehabilitation team focused on secondary prevention—such as medication, exercise, and lifestyle changes—could be an effective strategy. This approach would help redirect health care resources to those with a higher risk of adverse outcomes. Differences at baseline, such as patients in the MIMIC-IV database being more severely ill and having more comorbidities than those used for ANN training, may account for the ANN algorithm’s limited calibration. Similar phenomena were noted in the original article, which emphasizes that unbalanced training samples impact the performance of external validation, particularly in terms of calibration. To mitigate the negative effects of class imbalance on classifier training, rebalancing methods applied to the development cohort are essential [36]. In addition, implementing any model in clinical practice would require further recalibration for different sites [37]. Several approaches could improve the calibration of the ANN model: retraining the model using data more representative of the target population by reweighting the 21 variables, or revisiting the model’s input features to assess whether additional clinical indicators or biomarkers related to the prognosis of AMI could be incorporated to enhance the model’s accuracy.

In our study, the DCA demonstrated that the SWEDEHEART-AI model provided a significant net benefit in predicting 1-year mortality in patients with AMI when the risk threshold was below 19%. For instance, at a risk threshold of 15%, the SWEDEHEART-AI model identified 39.3 patients per 1000 who had a better net benefit compared with the no-treatment regimen (Multimedia Appendix 7). This suggests that the model can help identify patients early who may benefit from more aggressive secondary prevention strategies, such as closer monitoring, lifestyle modifications, and optimization of medication regimens. In clinical decision-making, selecting risk thresholds often requires balancing the benefits of an intervention against potential harms or resource constraints. A risk threshold of less than 19% indicates that the benefits of using the SWEDEHEART-AI model outweigh the potential harms or costs associated with false positives in this patient population. By contrast, the relatively low net benefit of the Qwen-2 and Llama-3 models suggests that these models may not be as effective in identifying high-risk patients without a significant increase in false positives. This aligns with clinical guidelines and underscores the importance of risk stratification to guide treatment decisions.

A review by Wessler et al [38] showed that many published cardiovascular prediction models had never been externally evaluated. For those that had, model performance tended to be overoptimistic due to the more simplified process in the external validation phase compared with the model development phase. External validation of SWEDEHEART-AI showed relatively good performance in predicting 1-year mortality in critically ill patients after MI. In our study, Qwen-2 and SWEDEHEART-AI exhibited almost equivalent clinical utility. Overall, Qwen-2 outperformed Llama-3, but both models require further refinement to achieve higher predictive performance in the future. The application of AI has demonstrated significant value in the prevention and management of cardiovascular disease, provided it is implemented within standardized, iterative clinical pathways [39-41]. Additionally, regulating the adoption of AI in medicine and health care is essential to ensure safety, maintain ethical standards, and protect patient privacy—an important yet challenging task [41].

### Study Limitations

This study has several limitations. First, our cohort was drawn from a critical care database in the United States, which may differ significantly from the SWEDEHEART-AI cohort derived from the Swedish population. Specifically, the 1-year all-cause mortality rate in our cohort was higher than that in the SWEDEHEART nationwide registry (475/2758, 17.22% vs 13,407/139,288, 9.63%). As a result, the SWEDEHEART-AI algorithm may not be entirely suitable for critically ill patients. Our critical care cohort limits the generalizability of our results. Implementing and assessing LLMs in clinical practice would require ongoing surveillance and potential recalibration to suit local environments. Further research is needed to explore the value of LLMs in other disease scenarios. Second, some records may have missing or incomplete data, which could affect the prediction accuracy of LLMs. Third, we used only 2 LLMs without fine-tuning or integrating structured data, which may have led to an underestimation of their capabilities. Directly asking LLMs to output numerical risks may overlook their inherent uncertainty (eg, probability distributions are not explicitly represented), potentially leading to unreliable results. AMI predictions involve a significant amount of medical expertise and domain-specific terminology that generic models may not adequately learn during their pretraining phase. Future work could enhance the performance of LLMs in the following directions: (1) exploring the engineering of cues for inputting longitudinal data (rather than single medical record data) into the LLM for prognostic prediction; (2) constructing multimodal models and integrating them into existing health care workflows to assess their utility in clinical decision-making; (3) domain-adaptive pretraining for medical texts; and (4) designing clinical task–oriented fine-tuning strategies (eg, reinforcement learning feedback). In addition, hybrid models that integrate SWEDEHEART-AI with LLMs can be explored to balance the advantages of structured and unstructured data.

### Conclusions

In this study, we evaluated the ANN algorithm and LLMs for predicting postdischarge 1-year all-cause mortality in patients following AMI from an intensive care cohort. The SWEDEHEART-AI score demonstrated good discrimination and clinical utility, and it may support improved clinical decision-making regarding treatment and follow-up planning in patients after AMI. Among the LLMs, Qwen-2 outperforms Llama-3 and demonstrates moderate predictive value. Qwen-2 and SWEDEHEART-AI show comparable classification effectiveness. In conclusion, we conducted an exploratory study on the ability of LLMs to predict mortality using a large volume of medical record data from critically ill patients. In the future, LLMs are expected to be integrated into clinical decision support systems to enable accurate risk assessment and stratification for patients with AMI. Further research is needed to optimize LLM performance and address calibration issues across diverse patient populations.

## Data Availability

The data used in this study are publicly available from the MIMIC-IV database [42] (access link). As a result of patient privacy concerns, the raw EHR text data cannot be shared publicly but can be accessed through a compliant application to MIMIC-IV.

## Appendix

Multimedia Appendix 1 Kaplan-Meier curves for all-cause mortality of risk grades based on SWEDEHEART-AI score.

Multimedia Appendix 2 Time-dependent receiver operating characteristics curves for all-cause mortality.

Multimedia Appendix 3 Precision-Recall curves for all-cause mortality.

Multimedia Appendix 4 Calibration column of SWEDEHEART-AI score.

Multimedia Appendix 5 Decision curve analysis for all-cause mortality.

Multimedia Appendix 6 Net benefit of using the 3 models for identifying 1-year all-cause death risk conditional on different risk thresholds.

Multimedia Appendix 7 Evaluate the incremental predictive value and predictive power of 3 models for 1-year mortality with net reclassification improvement and integrated discrimination improvement.

## Abbreviations

AI: artificial intelligence
AMI: acute myocardial infarction
ANN: artificial neural network
BERT: Bidirectional Encoder Representations from Transformers
DCA: decision curve analysis
DRG: diagnosis-related group
EHR: electronic health record
GRACE: Global Registry of Acute Coronary Events
IDI: integrated discrimination improvement
LLM: large language model
MIMIC: Medical Information Mart for Intensive Care
ML: machine learning
NPV: negative predictive value
NRI: net reclassification improvement
NSTEMI: non–ST-segment elevation myocardial infarction
PPV: positive predictive value
PRAISE: Prediction of Adverse Events Following Acute Coronary Syndrome
ROC: receiver operating characteristic
STEMI: ST-segment elevation myocardial infarction
SWEDEHEART: Swedish Web System for Enhancement and Development of Evidence-Based Care in Heart Disease Evaluated According to Recommended Therapies
TRIPOD: Transparent Reporting of a Multivariable Prediction Model for Individual Prognosis or Diagnosis
