# Supplementary figures and images for: Large Language Models and Artificial Neural Networks for Assessing 1-Year Mortality in Patients With Myocardial Infarction: Analysis From the Medical Information Mart for Intensive Care IV (MIMIC-IV) Database
Source: J Med Internet Res. 2025 May 12;27:e67253. doi: 10.2196/67253 (PMC12107198; doi:10.2196/67253)

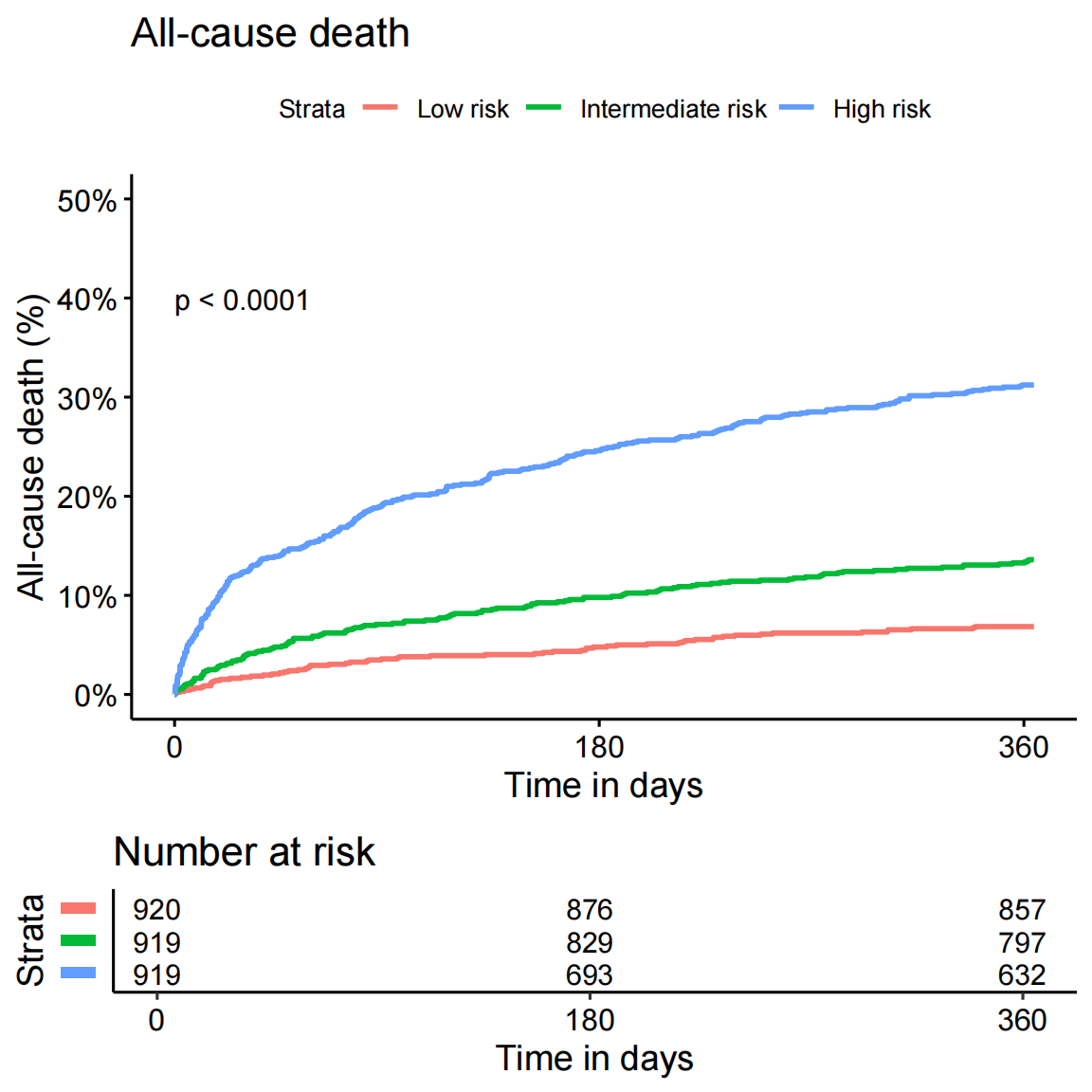

Supplement: Multimedia Appendix 1 [file jmir_v27i1e67253_app1.png]

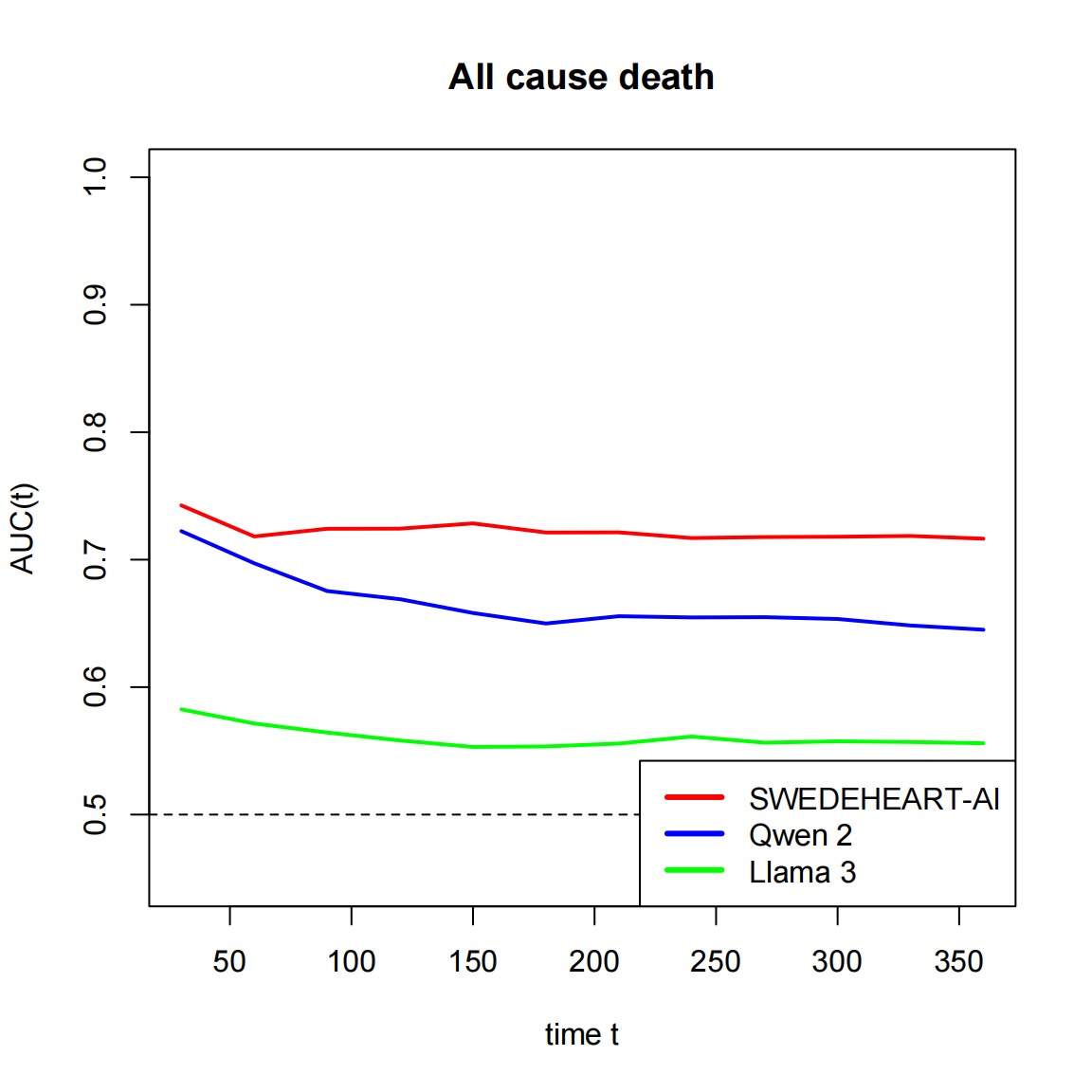

Supplement: Multimedia Appendix 2 [file jmir_v27i1e67253_app2.png]

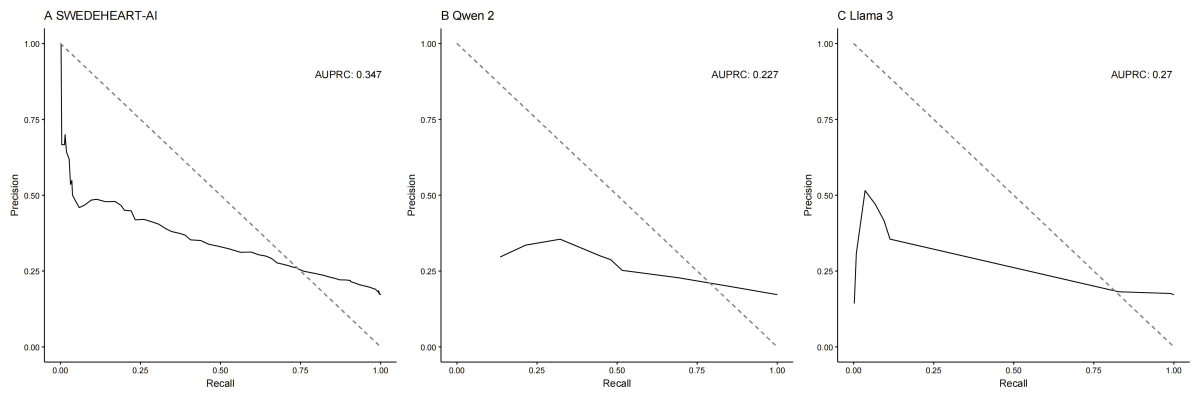

Supplement: Multimedia Appendix 3 [file jmir_v27i1e67253_app3.png]

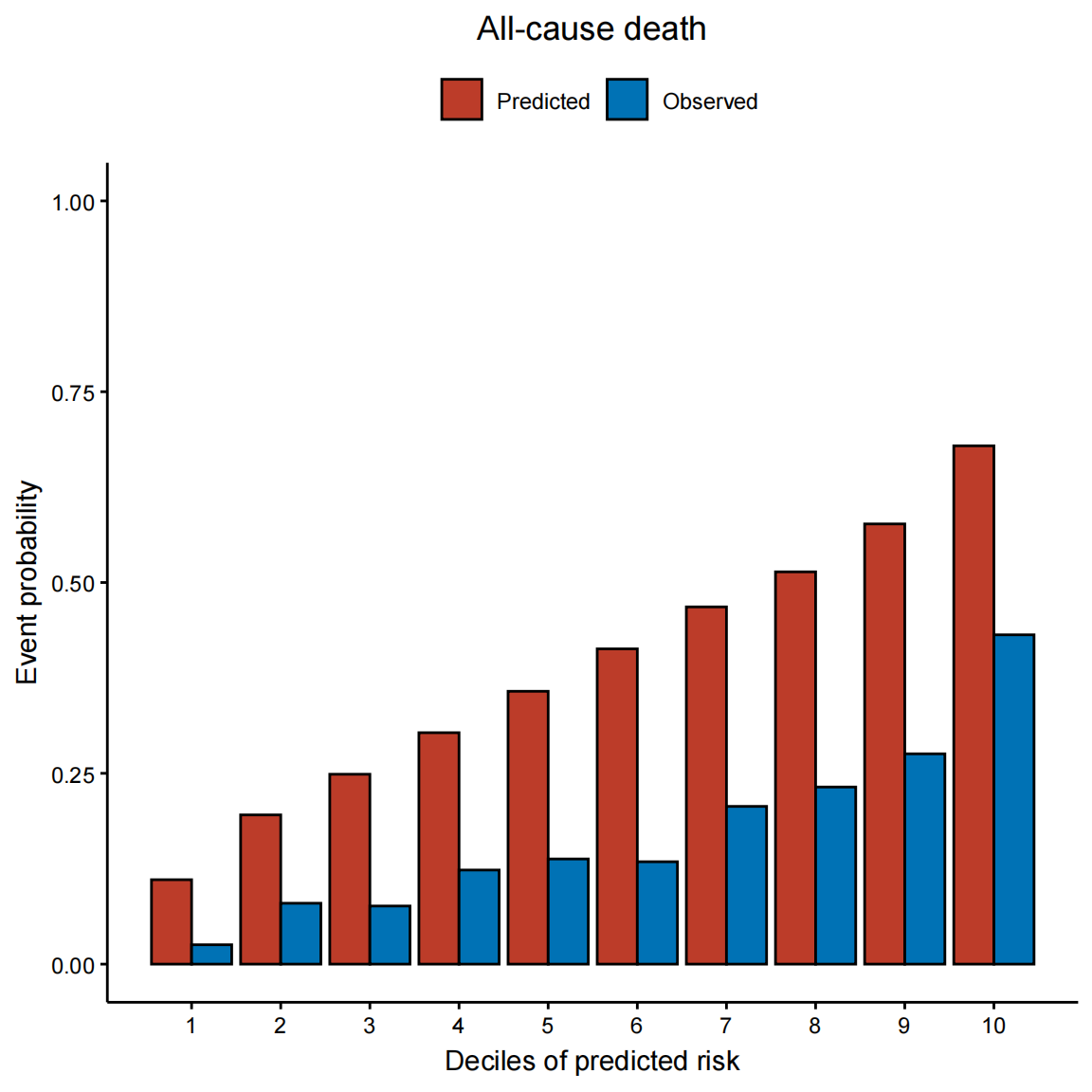

Supplement: Multimedia Appendix 4 [file jmir_v27i1e67253_app4.png]

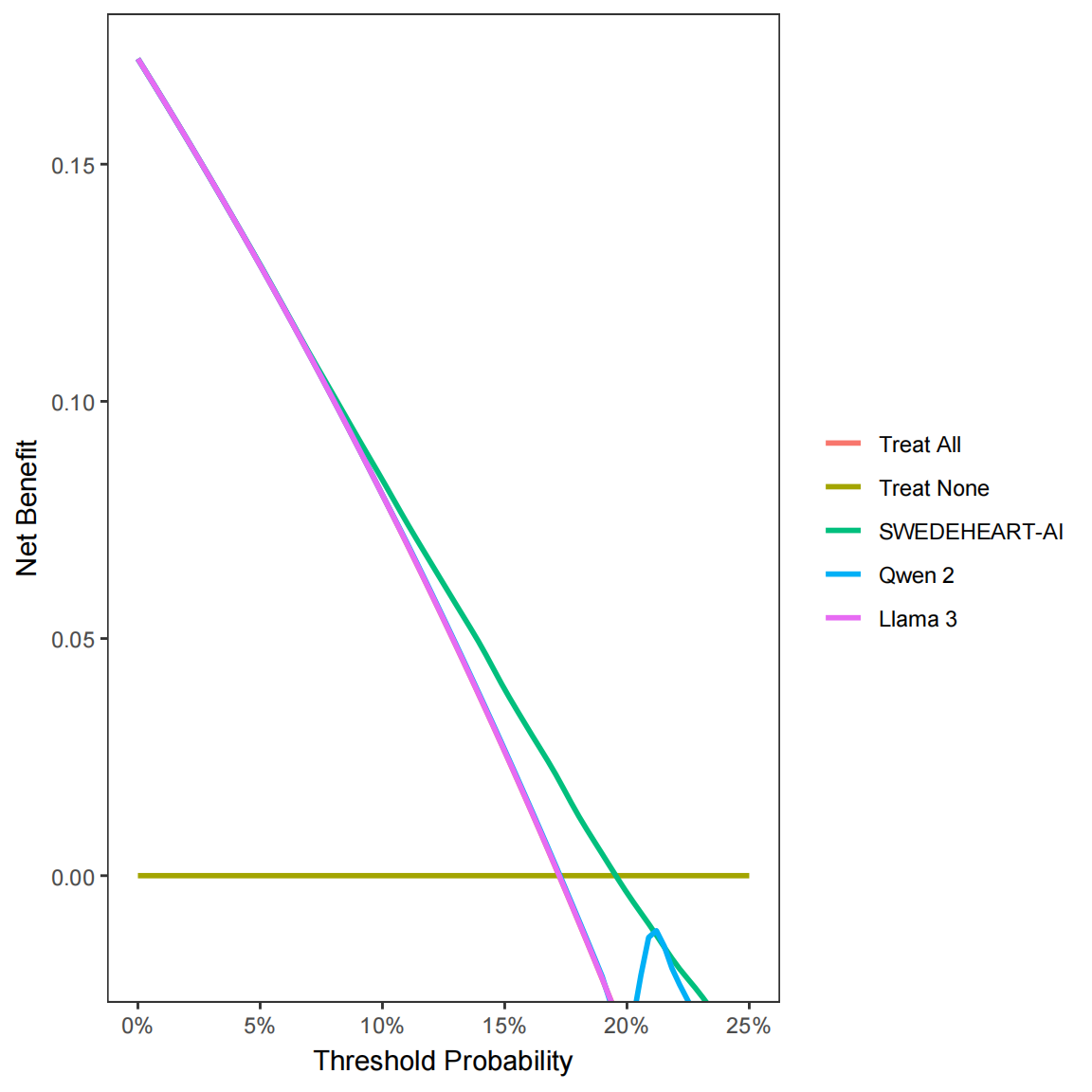

Supplement: Multimedia Appendix 5 [file jmir_v27i1e67253_app5.png]
