# Supplementary material for: Large Language Models and Artificial Neural Networks for Assessing 1-Year Mortality in Patients With Myocardial Infarction: Analysis From the Medical Information Mart for Intensive Care IV (MIMIC-IV) Database
Source: J Med Internet Res. 2025 May 12;27:e67253. doi: 10.2196/67253 (PMC12107198; doi:10.2196/67253)
Supplement: Multimedia Appendix 6 [file jmir_v27i1e67253_app6.docx]

| Risk threshold | Net benefit of treat none | Net benefit of treat all | Net benefit of SWEDEHEART-AI | Net benefit of Qwen-2 | Net benefit of Llama-3 |
| --- | --- | --- | --- | --- | --- |
| 5% | 0.00% | 12.87% | 12.88% | 12.87% | 12.87% |
| 10% | 0.00% | 8.03% | 8.34% | 8.03% | 8.03% |
| 15% | 0.00% | 2.61% | 3.93% | 2.65% | 2.62% |
| 20% | 0.00% | -3.47% | -0.36% | -3.42% | -3.46% |
| 25% | 0.00% | -10.37% | -3.78% | -5.23% | -9.55% |

Net benefit at different risk thresholds is calculated as: {true-positive classifications - [% risk threshold/ (100 - % risk threshold) × false-positive classifications]}/total number of participants.

The number of additional true positives per 100 patients that the risk scores can identify without additional false positives is calculated as follows: (net benefit of using the score of interest - net benefit of the alternative strategy in question)/ (% risk threshold / [100 - risk threshold]). This value is the equivalent to the reduction in false positives without a decrease in the number of true positives. For example, the number of true positives per 100 patients that the Qwen-2 can identify without additional false positives compared to the alternative strategy of assuming all as high risk, at a risk threshold of 2%, is: (0.30%--0.58%)/ (2%/ [100%-2%]) =43.1. SWEDEHEART-AI=Swedish Web system for Enhancement and Development of Evidence-based care in Heart disease Evaluated According to Recommended Therapies-Artificial Intelligence.
