# Supplementary material for: Large Language Models and Artificial Neural Networks for Assessing 1-Year Mortality in Patients With Myocardial Infarction: Analysis From the Medical Information Mart for Intensive Care IV (MIMIC-IV) Database
Source: J Med Internet Res. 2025 May 12;27:e67253. doi: 10.2196/67253 (PMC12107198; doi:10.2196/67253)
Supplement: Multimedia Appendix 7 [file jmir_v27i1e67253_app7.docx]

|  | **NRI or IDI** | ***P* value** |
| --- | --- | --- |
| SWEDEHEART-AI vs. Qwen-2 |  |  |
| Continuous NRI (95% CI) | -0.0247 (-0.1233, 0.0738) | 0.62 |
| Categorical NRI (95% CI) | 0.1259 (0.0600, 0.1919) | <.001 |
| IDI (95% CI) | -0.0089 (-0.0393, 0.0215) | 0.57 |
| SWEDEHEART-AI vs. Llama-3 |  |  |
| Continuous NRI (95% CI) | 0.4328 (0.3391, 0.5265) | <.001 |
| Categorical NRI (95% CI) | 0.2530 (0.2003, 0.3056) | <.001 |
| IDI (95% CI) | 0.1007 (0.0830, 0.1183) | <.001 |

Positive NRI values indicated more accurate reclassification, whereas negative values indicated more incorrect reclassification. Positive IDI values indicated improved discrimination and vice versa the model did not improve. NRI=net reclassification improvement. IDI=integrated discrimination improvement. SWEDEHEART-AI=Swedish Web system for Enhancement and Development of Evidence-based care in Heart disease Evaluated According to Recommended Therapies-Artificial Intelligence.
